# Elevated Serum α-Synuclein Autoantibodies in Patients with Parkinson’s Disease Relative to Alzheimer’s Disease and Controls

**DOI:** 10.3389/fneur.2017.00720

**Published:** 2017-12-22

**Authors:** Ali Shalash, Mohamed Salama, Marianne Makar, Tamer Roushdy, Hanan Hany Elrassas, Wael Mohamed, Mahmoud El-Balkimy, Mohamed Abou Donia

**Affiliations:** ^1^Department of Neurology, Faculty of Medicine, Ain Shams University, Cairo, Egypt; ^2^Medical Experimental Research Centre (MERC), Faculty of Medicine, Mansoura University, Mansoura, Egypt; ^3^Faculty of Medicine, Toxicology Department, Mansoura University, Mansoura, Egypt; ^4^Faculty of Medicine, Okasha Institute of Psychiatry, Ain Shams University, Cairo, Egypt; ^5^Faculty of Medicine, Department of Clinical Pharmacology, Menoufia University, Shebin El-Kom, Egypt; ^6^Basic Medical Science Department, Kulliyyah of Medicine, International Islamic University Malaysia, Kuantan, Malaysia; ^7^Department of Pharmacology and Cancer Biology, Duke University Medical Center, Durham, NC, United States

**Keywords:** autoantibodies, biomarkers, α synuclein, Parkinson’s disease, Alzheimer’s disease

## Abstract

Early diagnosis of neurodegenerative diseases is of paramount importance for successful treatment. Lack of sensitive and early biomarkers for diagnosis of diseases like Parkinson’s disease (PD) is a handicapping problem for all movement disorders specialists. Using serum autoimmune antibodies (AIAs) against neural proteins is a new promising strategy to diagnose brain disorders through non-invasive and cost-effective method. In the present study, we measured the level of AIAs against α-synuclein (α-syn), which is an important protein involved in the pathogenesis of PD. In our study patients with PD (46 patients), Alzheimer’s disease (AD) (27 patients) and healthy controls (20 patients) were evaluated according to their sera α-syn AIAs levels. Interestingly, α-syn AIAs were significantly elevated in PD group compared to AD and healthy controls, which advocates their use for diagnosis of PD.

## Introduction

Dementia of Alzheimer’s type and Parkinson’s disease (PD) are considered the first and the second most common neurodegenerative disorders worldwide within adults. The prevalence of Alzheimer’s disease (AD) is 1–2% at the age of 65 years, which doubles every 5 years to >35% at the age of 85 years, while the PD prevalence is 1% over 65 years and reaches 4% over 80 years (1, 2).

Parkinson’s disease is characterized by the loss of dopaminergic neurons in the substantia nigra pars compacta (SNc) and the accumulation of insoluble cytoplasmic protein inclusions (Lewy bodies), which is composed of α-synuclein (α-syn) (1, 3). Thus, α-syn aggregation is a central component of the pathogenesis of PD, which interferes with different cellular functions including lysosomal and mitochondrial functions, autophagy, vesicular homeostasis, and microtubule transport, and induces neuroinflammatory process (4). Additionally, α-syn aggregation has a role in the pathogenesis of AD through its interaction with tau protein and amyloid and has been used as potential biomarker by previous studies (5–7). Accordingly, some studies investigated the α-syn autoantibodies (AIAs) as putative biomarker compared patients with PD, to patients with AD along with controls (8).

Furthermore, several animals and patients’ studies confirmed the connection between neuroinflammation and neurodegenerative disorders through activation of microglia and astrocytes. Moreover, humoral immunity has an integrative role in PD pathogenesis, which might prevent further progression (9). Subsequently, the inflammatory and immune mediators have been investigated as potential biomarkers (3, 10).

Discovering biomarkers for confirming diagnosis and/or determining progression of these neurodegenerative disorders was the target of several recent trials. A working group of National Institute of Health defined the biomarker as “a characteristic that is objectively measured and evaluated as an indicator of normal biologic processes, pathogenic processes, or pharmacologic responses to a therapeutic intervention” (11). Ideal biomarker should be linked to neurodegeneration mechanisms, specific, reproducible, non-invasive, easy to use, and inexpensive (12).

In neurodegenerative disorders, naturally occurring AIAs are produced by the immune system against released disease-associated proteins or their fragments into circulation from regions of ongoing pathological changes and cell death. Thereafter, AIAs bind to the disease-associated debris in blood and could gain access to the brain to bind to their related antigens (13).

Consequently, several studies explored the levels of α-syn or its AIAs in patients’ cerebrospinal fluid (CSF) (5, 14, 15) and/or plasma (16), with contradictory results (17). Recently, several studies reported association of α-syn and cognitive impairment in PD patients (16). Despite the plasma studies showed more inconsistency, the non-invasive nature, lower costs, and the advances of methodological methods maintained the ongoing exploration of the plasma biomarkers (3, 10).

Exploring biochemical biomarkers is of remarkable importance in chronic neurodegenerative disorders such as PD and AD. They could improve diagnostic accuracy in early stages, distinguish both diseases with overlapping symptoms, develop disease modifying treatments, and explore novel molecular neuropathological processes (5).

Thus, the aim of this study was to investigate the value of the naturally occurring serum autoantibodies of α-syn protein as potential biomarker for diagnosis of PD in comparison to AD and healthy controls.

## Materials and Methods

A total of 93 individuals were enrolled in the study between June 2016 and June 2017 after approval through a written formal consent and after receiving the approval of the scientific ethical committee of Faculty of Medicine—Ain Shams University.

The study was composed of three groups, 27 clinically confirmed cases of dementia of Alzheimer’s type, 46 clinically confirmed cases of PD, and 20 healthy controls. All participants were subjected to complete medical history, brain imaging, and basic laboratory investigations to guard against any other possible cause serving their manifestations. PD patients, diagnosed according to the British Parkinson’s Disease Society Brain Bank criteria, were included (18) by a consultant neurologist from Ain Shams University, Movement Disorders Clinic, Cairo, Egypt. Patients with PD were assessed using the Unified Parkinson’s Disease Rating Scale (UPDRS), Hoehn and Yahr scale (H&Y), and Schwab and England scales (S&E) in “medication Off” and “On” states. Exclusion criteria of parkinsonian group included the presence of dementia, atypical or secondary parkinsonism, and familial parkinsonism.

Patients with AD were recruited from outpatients clinic of department of Neurology and institute of psychiatry, Ain Shams University, diagnosis according to the NINCDS-ADRDA and DSM-IV criteria for dementia (19) and assessed using the Arabic version of the Montreal Cognitive Assessment (MoCA) test (20, 21).

Following confirmation of diagnosis, blood samples were withdrawn, centrifuged immediately, sera were obtained from collected blood samples, and stored in −20 freezers prior to their collection in Biobank. All collected sera were stored eventually in −80 freezer of the Medical Experimental Research Center (MERC) of Mansoura University.

### Autoantibodies Estimation

IgG anti α-syn antibodies have been tested using commercially available enzyme-linked immunosorbent assay (ELISA) kit purchased from MyBioSource Inc. (San Diego, CA, USA; Cat. No. MBS 2086950). Testing steps have been carried out according to the manufacturer’s provided protocols. The titers were estimated on the base of calibration curve of autoantibody standards and expressed in nanograms per milliliter (ng/ml). The sensitivity in the assay was 0.1 ng/ml.

### Data Analysis

The collected data were revised, coded, tabulated, and introduced to a PC using Statistical package for Social Science (SPSS 20). Data were presented and suitable analysis was done according to the type of data obtained for each parameter as descriptive study of the three different groups, followed by comparative, correlation and regression studies.

Analysis was performed to identify the ability of autoantibodies to separate cases (PD and AD) from controls. Moreover, further analysis was made to find a differentiation threshold between different neurodegenerative diseases, e.g., PD from AD (in our case). Analyses included: correlation value for each marker, histograms for levels of each autoantibody in different categories (controls, PD, and AD) and finally distribution level analysis. Correlations were calculated for serum levels of α-syn autoantibodies (AIAs) with clinical data of studied groups using Pearson’s (for parametric data) and Spearman’s (for non-parametric data) coefficients tests.

## Results

Group characteristics of study populations are shown in Table 1. The mean age of parkinsonian subgroup was 56.26 ± 12.26 years (range 29–81 years), and of Alzheimer’s subgroup was 70.07 ± 8.31 years (range 54–81 years), while the mean age of the control group was 53.95 ± 10.65 years (range 33–72 years). There was no statistical significant difference between both the control and parkinsonian subgroup (*p* = 0.431), while there is high statistical significance between the control group and Alzheimer’s subgroups (*p* < 0.001) and the parkinsonian and Alzheimer’s subgroups (*p* < 0.001).

**Table 1:** Demographic and clinical data of the three studied groups.

	Parkinson subgroup	Alzheimer’s subgroup	Control group

	No. = 46	No. = 27	No. = 20
Gender; males (%)/females (%)	23 (50.0%)/23 (50.0%)	15 (75.0%)/5 (25.0%)	12 (44.4%)/15 (55.6%)
Age; mean ± SD (range)	56.26 ± 12.26	70.07 ± 8.31	53.95 ± 10.65
	29–81	54–81	33–72
Duration of illness; mean ± SD (range)	5.20 ± 3.36 (0.5–15)	4.0.46 ± 2.54 (1–9)	
Age of onset; mean ± SD (range)	51.07 ± 12.18 (25–79)		
H&Y—Off; median (range)	3 (1–5)		
H&Y—On; median (range)	1 (1–2)		
S&E—Off; mean ± SD (range)	54.75 ± 25.22 (10–90)		
S&E—On; mean ± SD (range)	85.00 ± 17.05 (40–100)		
Unified Parkinson’s Disease Rating Scale (UPDRS) I Off; mean ± SD (range)	4.58 ± 2.93 (0–11)		
UPDRS III Off; mean ± SD (range)	43.00 ± 19.91 (8–77)		
Montreal Cognitive Assessment; mean ± SD (range)		16.63 ± 5.41 (7–25)	
Serum α-syn autoantibodies (ng/ml); median (range)	4.23 (3.3–5.63)	2.9 (1.2–3.3)	0.46 (0.07–0.93)

The mean duration of illness in parkinsonian patients was 5.20 ± 3.36 years and in Alzheimer’s patients was 4.46 ± 2.54 years. The median of H&Y—Off was 3 (1.5–3.5), 75% of patients was in stage 1–3, and the mean of S&E—Off was 54.75 (±25.22) The mean of MOCA test for Alzheimer’s patients was 16.63 ± 5.41 (11–22; Table 1).

### α-Syn AIAs, α-syn Autoantibodies

**Figure 1:**
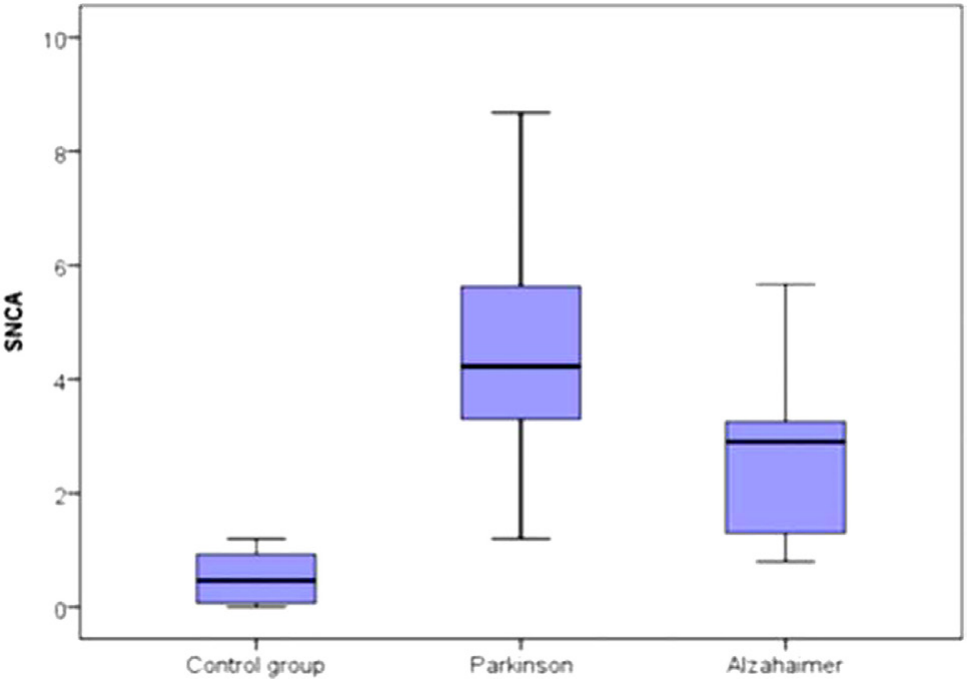
Comparison between the three studied groups regarding the median of serum α-synuclein autoantibodies.

The median of serum α-syn AIAs was highest in patients with PD [4.23 ng/ml (3.3–5.63)], and elevated in Alzheimer’s patients [2.9 ng/ml (1.2–3.3)] less than PD patients, while lowest in controls [0.46 ng/ml (0.07–0.93)]. On comparing these levels of the three studied groups, there was high statistical significant difference (*p* < 0.001) (Figure 1).

Within PD group, patients elder than 60 years (18 patients) had significantly higher serum α-syn antibodies compared to patients younger than 60 years (*p* = 0.037). Furthermore, PD cohort was divided to two subgroups according to disease staging, <3 (17 patients) and ≥3 (23 patients). Patients with milder stages had non-significantly higher serum α-syn AIAs (4.94 ± 2.61) compared to more advanced stage (4.29 ± 1.80), while both were significantly higher than controls (*p* < 0.001). There was no significant difference between PD patients of duration <5 years (25 patients, 4.62 ± 2.08) and ≥5 years (21 patients, 4.84 ± 2.34) (*p* = 0.739). There were no significant differences between males and females within each subgroup.

**Figure 2:**
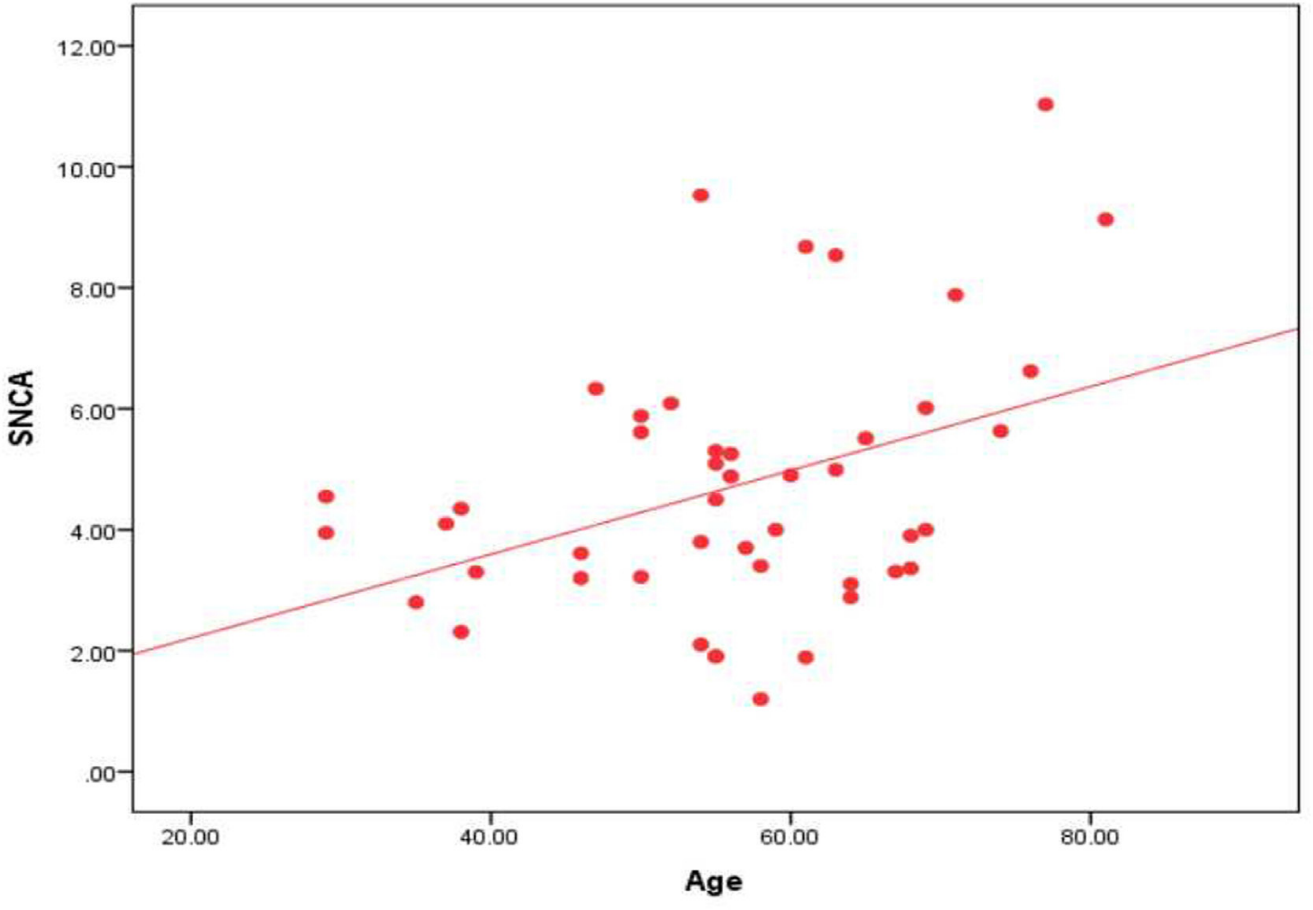
Correlation between the level of α-syn autoantibodies and age in the Parkinson’s subgroup.

In the Parkinson’s subgroup, there was significant correlation between the serum level of α-syn AIAs and age of patients (*r =* 0.390, *p* = 0.007) and age of onset (*r* = 0.383, *p* = 0.009) (Figure 2), while there were no significant correlations with disease stage or UPDRS cognition and motor sub-scores. There were no significant correlations in the Alzheimer’s subgroup.

**Figure 3:**
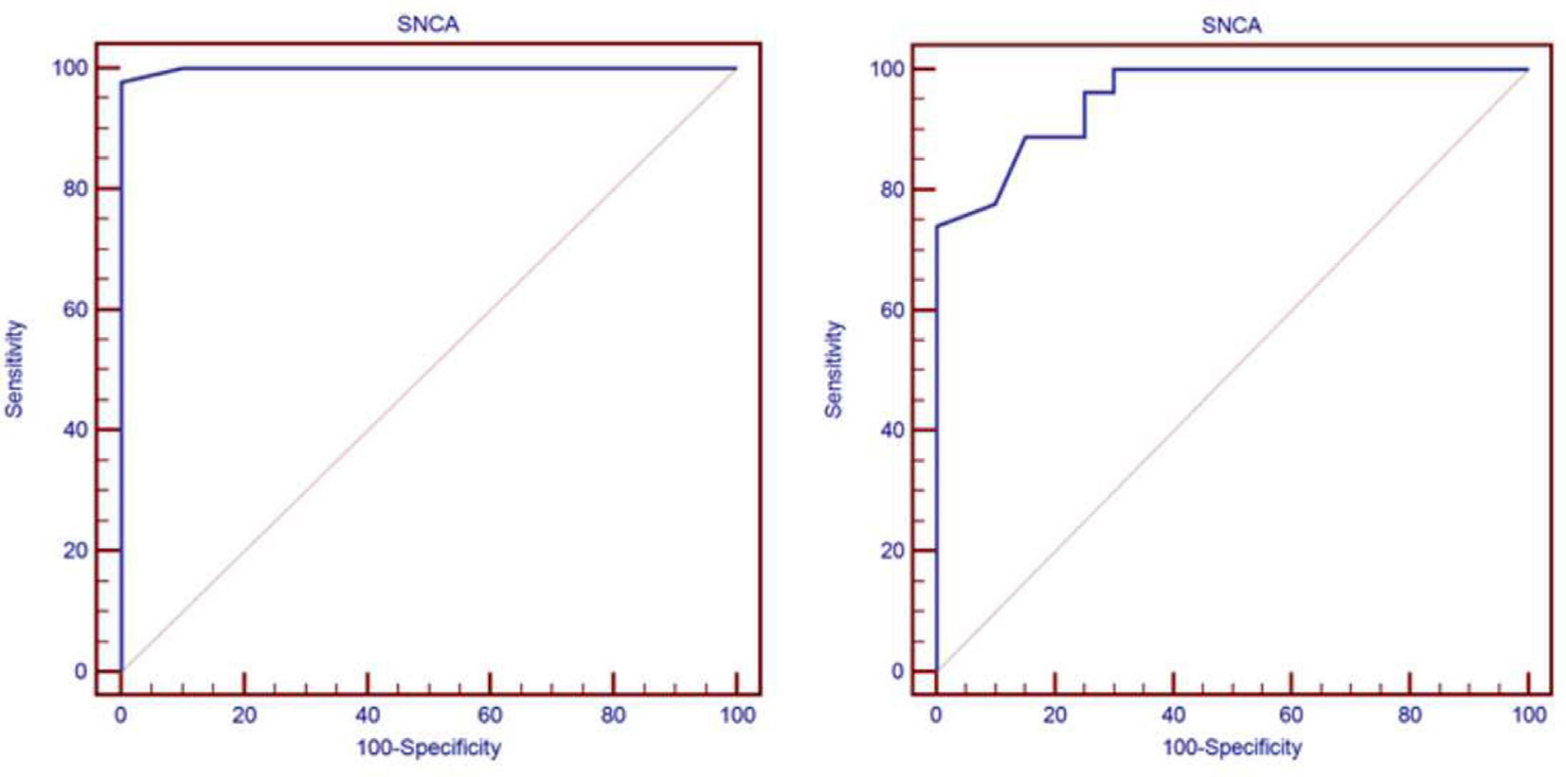
Roc curve of predicting Parkinson’s and Alzheimer’s groups.

On further analysis of the serum level of α-syn AIA as a predictor biomarker in Alzheimer’s and PD using Roc curve. In the Alzheimer’s subgroup, the cutoff point of α-syn AIA was >1.2, its AUC was 0.955, its sensitivity reached 74.07, and its specificity was 100.00. In the Parkinson’s subgroup, its cutoff point was >1.2, its AUC was 0.999, its sensitivity reached 97.83, and its specificity was 100.00 (Figure 3).

## Discussion

The inconsistency and infrequency of studies that explored anti α-syn AIAs in serum of PD patients warranted further studies of this non-invasive and inexpensive biochemical biomarker (3). The current study investigated the serum anti α-syn AIAs as potential biomarker in patients with PD compared with AD and healthy controls. Remarkably, this study found significantly elevated serum anti α-syn AIAs in PD patients more than AD patients and healthy controls, with high sensitivity and specificity of cut off level >1.2 as predictor biomarker (97.83 and 100%, respectively). Additionally, the serum anti α-syn AIAs was significantly elevated in AD patients compared to healthy controls.

Similar findings were reported by prior studies. Horvath and his colleagues also found that the titers of anti α-syn AIAs in blood samples of 60 recently diagnosed parkinsonian patients was higher compared to age-matched controls, which was correlated also to CSF α-syn AIAs (22). Likewise, other prior studies also demonstrated higher serum α-syn AIAs versus controls (23). However, they reported return of their titer to controls level with longer duration (24).

In contrast to the current study, other studies reported comparable (25–27) or lower (8, 28, 29) serum titer of α-syn AIAs compared to controls. Maetzler et al. reported comparable serum α-syn AIAs in 93 PD patients compared to controls; however, their study was of elder age, longer duration, and non-age-matched controls (25). Besong-Agbo et al. reported lower level of serum α-syn Abs in PD patients than patients with AD and controls (8). They attributed their findings to advanced stage of their PD cohort, methodological differences, and low avidity of naturally occurring Abs (8). Recently, Brudek and his colleagues have investigated the apparent affinity of anti-α-syn AIAs in plasma samples from 46 PD patients and 41 controls using ELISA and found that the occurrence of high affinity anti-α-syn AIAs in plasma from PD patients is reduced compared to healthy controls (28).

Variability of findings of previous studies could be attributed to methodological and patients’ characteristics variability (3, 8, 27). Avidity of natural autoantibodies was characterized as methodological causes of variability (8). Patients’ variability included different age’s range, duration, severity, and genetic inheritance (22, 25). Noteworthy, the current study is characterized by younger age and shorter duration compared to other studies.

Several prior studies demonstrated higher serum α-syn AIAs in patients with shorter duration (9, 23, 24). In contrast to these studies and in accordance to another studies (8, 25), this study did not show correlation with disease duration. However, we detected non-significant decline of serum α-syn AIAs with more advanced stages, in accordance to the recent study by Horvath et al. (22). In this study, the serum α-syn AIAs was correlated to age of PD patients. Other studies did not detect association with age (8, 22, 25). Furthermore, we could not detect correlation between and disease stage (H&Y scores), similar to other studies (8, 25, 27).

Recently, despite using different approaches, El-Agnaf group revealed significant elevation of α-syn in PD patients’ CSF (27) with parallel decline in their plasma level (28). The elevated levels of protein in CSF with their decline in plasma—in accordance to our findings—suggest a possible role for AIAs in clearance of α-syn from sera of PD patients compared to controls.

On the other hand, this study detected elevated α-syn AIAs in serum of patients with AD, compared to controls, in concur with previous studies (7, 9, 30). However, it was lower than PD cases. Moreover, recently, serum α-syn was higher in PD, and correlated with cognitive decline, rather than motor severity (16). Although previous reports suggested possible interaction between tau, B-amyloid, and α-syn levels (30, 31), further studies are needed to confirm a possible contribution of α-syn in AD. The present findings support our previous studies on identification of AIAs against different cytoskeletal proteins in brain insult (32, 33).

## Conclusion

The present study showed that serum level of AIAs against α-syn could help as biomarker for PD as they could identify PD patients compared to healthy controls and patients with other neurodegenerative disease (AD). Moreover, our work showed that α-syn AIAs level were higher in AD patients compared to healthy controls, which suggest possible role for α-syn in AD that need to be studied in future research. Further studies are warranted to reproduce these findings, investigating larger number of patients, differentiating types of PD, distinguishing younger onset and classic types, and examining different disease severity and durations.

## Ethics Statement

The study was conducted after approval through a written formal consent and after receiving the approval of the scientific ethical committee of Faculty of Medicine—Ain Shams University and IRB of Faculty of Medicine, Mansoura University.

## Author Contributions

AS: concept, study design, data generation, data analysis, and writing manuscript. MS: hypothesis, concept, study design, data generation, data analysis, and writing manuscript. MM: data generation. TR: data analysis and critical review. HE: data generation, data analysis, and drafting manuscript. WM: study design and data analysis. ME-B: data generation, data analysis, and critical review. MA-D: hypothesis, concept, study design, and critical review.

## Conflict of Interest Statement

The authors declare that the research was conducted in the absence of any commercial or financial relationships that could be construed as a potential conflict of interest.
