# Supplementary material for: Elevated Serum α-Synuclein Autoantibodies in Patients with Parkinson’s Disease Relative to Alzheimer’s Disease and Controls
Source: Front Neurol. 2017 Dec 22;8:720. doi: 10.3389/fneur.2017.00720 (PMC5744443; doi:10.3389/fneur.2017.00720)
Supplement: Supplementary file 1 [file Table_1.docx]

**Table A Supplementary: Correlation between age of onset, duration of illness and the severity of illness with the level of** α-syn**AIAs in the Alzheimer’s and the Parkinson subgroups:**

|  | **Alzheimer’s subgroup** | | **Parkinson subgroup** | |
| --- | --- | --- | --- | --- |
|  | **SNCA** | | | |
|  | **r** | **P-value** | **r** | **P-value** |
| Age | -0.144 | 0.475 | *0.390* | ***0.007*** |
| Duration of illness | 0.255 | 0.199 | 0.028 | 0.852 |
| Age of onset |  |  | *0.383* | ***0.009*** |
| H&Y- off | - | - | -0.184 | 0.255 |
| H&Y -on | - | - | 0.014 | 0.933 |
| S&E- OFF | - | - | -0.007 | 0.967 |
| S&E -ON | - | - | 0.100 | 0.551 |
| UPDRS I-Off | - | - | -0.159 | 0.322 |
| UPDRS III-Off | - | - | -0.261 | 0.114 |
| MOCA | 0.217 | 0.278 | - | - |
